# A hybrid machine learning/deep learning COVID-19 severity predictive model from CT images and clinical data

**DOI:** 10.1038/s41598-022-07890-1

**Published:** 2022-03-14

**Authors:** Matteo Chieregato, Fabio Frangiamore, Mauro Morassi, Claudia Baresi, Stefania Nici, Chiara Bassetti, Claudio Bnà, Marco Galelli

**Affiliations:** 1 Unit of Medical Physics, Fondazione Poliambulanza Istituto Ospedaliero, 25124 Brescia, Italy; 2 Department of Diagnostic Imaging, Unit of Radiology, Fondazione Poliambulanza Istituto Ospedaliero, 25124 Brescia, Italy; 3 Unit of Lean Managing, Fondazione Poliambulanza Istituto Ospedaliero, Information and Communications Technology, 25124 Brescia, Italy; 4 Present Address: Tattile s.r.l, 25030 Mairano, BS Italy; 5 Present Address: Unit of Medical Physics, Spedali Civili, 25124 Brescia, Italy

**Keywords:** Medical research, Infectious diseases

## Abstract

COVID-19 clinical presentation and prognosis are highly variable, ranging from asymptomatic and paucisymptomatic cases to acute respiratory distress syndrome and multi-organ involvement. We developed a hybrid machine learning/deep learning model to classify patients in two outcome categories, non-ICU and ICU (intensive care admission or death), using 558 patients admitted in a northern Italy hospital in February/May of 2020. A fully 3D patient-level CNN classifier on baseline CT images is used as feature extractor. Features extracted, alongside with laboratory and clinical data, are fed for selection in a Boruta algorithm with SHAP game theoretical values. A classifier is built on the reduced feature space using CatBoost gradient boosting algorithm and reaching a probabilistic AUC of 0.949 on holdout test set. The model aims to provide clinical decision support to medical doctors, with the probability score of belonging to an outcome class and with case-based SHAP interpretation of features importance.

## Introduction

**Figure 1:**
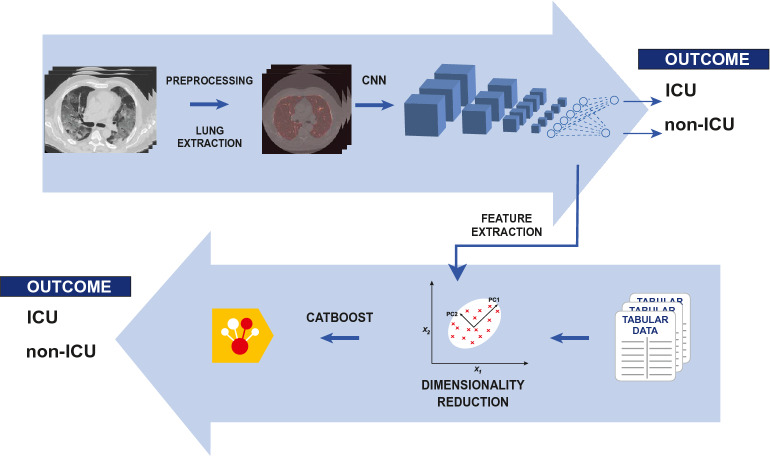
A graphical representation of the proposed model.

To date (May 2021), more than one hundred millions of individuals have been reported as affected by COVID-19. More than two millions deaths have been ascribed to the infection. All over the world, the sheer numbers of the pandemic pose a heavy burden on emergency departments, hospitals, intensive care units and local medical assistance. From the beginning of the infection, it was apparent that COVID-19 encompasses a wide spectrum of both clinical presentations and consequent prognosis, with cases of sudden, unexpected evolution (and worsening) of the clinical and radiological picture^1^. Such elements of variability and instability are still not fully explained, with an important role advocated for a multiplicity of pathophysiological processes^2–4^. In this context, it would be natural to try to exploit techniques of artificial intelligence, fueled by the availability of large data amounts, to support clinicians. Indeed, a large number of efforts in this sense has already been done, headed on different tasks, in particular diagnosis and prognosis^5,6^. We focused on the latter, taking into account in particular clinical usability. We defined as our goal to build an hybrid machine learning/deep learning severity predictive model that can act as an auxiliary tool for patient risk assessing in clinical practice. In order to accomplish the objective, we considered essential the combination of imaging and non-imaging data. We chose to exploit a 3D Convolutional Neural Network (CNN) as feature extractor, and CatBoost, a last generation gradient boosting model, as classifier of tabular data^7,8^. The proposed model is represented graphically in Fig. 1. The output of the model is both the percentage score of the outcome and the SHAP (SHapley Additive exPlanations) evaluation of feature importance in the individual prediction^9,10^. The SHAP libraries allow to calculate feature importance in each patient prediction as game theoretical Shapley values. In this framework, the prediction is the game. Features are players, and the prediction result is the payout of the game. The Shapley values are a fair distribution of the payout between players, i.e. of the prediction result between features. In this way, both synthetic (percentage score) and analytic (SHAP values) information are provided to the judgement of the clinician.

### Related works

Machine learning and deep learning methods have been applied to model prognosis of COVID-19 patients starting from clinical and laboratory data, imaging data (chest X-rays, CXR, computed tomography, CT, ultrasound) or a combination of both.

A quite large number of works utilized only tabular data. Clinical records availability allowed the gathering of large datasets, often of some thousands (see the review of^11^).

A substancially smaller number of works based prognosis on imaging information only (e.g.^12–15^).

However, since from the start of the pandemics, it has been recognized the importance of the role played by inflammation and by the systemic immunitary response^4^. Furthermore, multi-organ involvement is often found in critical patients^2^. Hence, the need of integrated information besides lungs imaging, an approach to COVID-19 prognosis closer to the present work. A non-exhaustive list of works follows (see also^6^).

Zhang et al.^16^ built models that performed segmentation of lesions, diagnosis and prognosis. Starting from 4965 annotated CT slices of COVID-19 and other pneumonia affected patients, they tested different 2D architectures for seven classes segmentation, and finally adopted a DeeepLabv3 architecture. For diagnosis, a classification model with an adapted ResNet3D architecture is then built on the top of the stacked 2D lesion maps (that is, a seven channel one hot encoded downsampled CT volume), using 2507 patients. Finally, volumes, densities and ratios obtained from the lesion map (e.g. ground-glass opacities volume, consolidation volume) are feed along with clinical and laboratory data in a Cox proportional hazards regression and in a LightGBM machine learning model for prognosis, in a cohort of 456 hospitalized patients. The chosen prognostic outcome is death or ICU admission or mechanical ventilation, and the obtained AUC for the integrated model is 0.91 (five-fold cross-validation).

Starting from deep learning segmentation, some authors used radiomic features for prognosis.

Chassagnon et al.^17^ used an ensemble of deep learning architectures for lesion segmentation. After a thorough feature selection analysis, they selected 5 radiomic features from the lesion volume, 5 from the heart region, 5 from the lung region, and added disease extent and some clinical features. From these 23 features, they built an ensemble machine learning model to predict a three classes outcome (short term deceased, long term deceased, long term recovered). Their cohort consisted of 693 patients, and the reported balanced accuracy is 0.71 on the holdout test (for separate binary outcomes, the reported AUC ranges from 0.76 to 0.86).

Chao et al.^18^ used a pretrained 2D U-Net for lung and lobes segmentation, and a hierarchical encoder-decoder architecture for lesion segmentation. For prognosis, they built a random forest with radiomic features extracted both from the whole lung and the lesion volume, and laboratory/clinical data. They operated on three separated dataset, with different laboratory data, of respectively 117, 125 and 57 patients. For the ICU admission endpoint, they reported AUC ranging from 0.84 to 0.88 in five-fold cross validation in each of the datasets.

Wu et al.^19^ segmented CT volumes with a pretrained 3D DenseNet. With radiomics and clinical features (not laboratory), they predicted death or ICU admission or the need for mechanical ventilation with a Fine-Gray competing risk regression. Their cohort is 492 patients, and the reported AUC is respectively 0.86 and 0.98 in external validation, for patients with CT scan performed before and after a week from symptoms onset.

Other works combined the results from a deep learning classifier on imaging data with those from a different classifier on clinical/laboratory variables.

Ning et al.^20^ utilized a 13 layer 2D CNN to select the ten most significant CT slices for each patients. They then combined the results of another 2D 13 layers CNN on the selected slices with those of a 7 layers neural network on clinical and laboratory data, using a penalized logistic regression. Outcomes were assessing morbidity and predicting mortality. Their dataset consists of 1522 patients (controls included) for the first task, 719 for the second. In the mortality prediction , their model had an AUC of 0.856.

Lassau et al.^21^ stacked two pretrained CNN (EfficientNet B0 and ResNet50) to predict severity from CT scans. The result was then feed in a penalized logistic regression alongside with five selected clinical variables. The chosen endpoint were ’$O_{2}\ge$15 L/min or ventilation or death’, ’ventilation or death’ and death. AUC obtained on the external test were respectively 0.79, 0.86 and 0.88. The cohort of patients for the severity task amounted to 931.

Jiao et al.^22^ and Wang et al.^23^ used the same method, i.e. they combined results of an EfficientNet on images and a neural network on clinical data to predict severity (death, ICU, need of mechanical ventilation). They also predicted time to adverse event combining results of two survival forests, one on clinical data, the other on 256 features extracted from the ImageNet. They modeled respectively a dataset of 2309 patients with CXR and a dataset of 1051 patients with CT (ten sliced selected for the prognostic assessment). Results for the severity classification were AUC 0.846 for the CXR set, AUC 0.83 for the CT set, for the time to adverse event for the CXR set C-index 0.805, for the CT set 0.801.

Shamout et al.^24^ predicted deterioration at 96 hours from CXR and clinical data of 3061 first care patients. Their model reached an AUC of 0.786, and consists of ensembled deep learning and LightGBM models respectively on CXR and clinical data.

An handful of works combined clinical data and imaging at a lower level, creating a joint model. The two well known methods are to inject at some point of a deep learning model the tabular clinical data, or to use extracted deep learning features as tabular data in a (traditional) machine learning model.

Kwon et al.^25^ injected clinical data from the emergency department in the last layer (fully connected) of a DenseNet-121 architecture. They predicted 30-day intubation and death outcomes, with AUC respectively of 0.88 and 0.82, from a 499 patients cohort.

Similarly, Ho et al.^26^ concatenated 19 clinical features at the classifying fully connected layer of a 3D CNN operating on CT volumes. They predicted adverse event (including death and ICU admission) in a 297 patients dataset, with AUC 0.916 on five fold cross validation.

Xu et al.^27^ extracted 10 features from a 2D CNN (a customized ResNet architecture) working on CT slices and combined them with 23 clinical and 10 laboratory features in some traditional machine learning methods (random forest, support vector machine, LASSO) to assess patient membership to one of four class: mild COVID-19, severe COVID-19, other pneumonia, healthy. Their dataset consisted of 689 patients (362 with COVID-19). The accuracy obtained in the test set for the three machine learning methods ranged from 95.4 to 97.7%.

Fang et al.^28^ used a 1040 patients dataset to build a model from multiple time points CT scans and clinical variables. A CNN is used as feature extractor for each time point CT scan. Clinical data are processed by a multi layer perceptron. Features from time points and last perceptron layer are used as input of a predicting long short term memory (LSTM). The outcome is the prediction of malignant progression, identified by death, ICU admission for organ failure or deterioration of respiratory indices. The AUC obtained by the model is 0.885 on a same hospital test cohort, but drops to 0.651 in a different hospital cohort.

Soda et al.^29^ built three models on a 820 patients dataset with CXR and clinical data. First and second model are traditional machine learning models. First model input are handcrafted features alongside with cinical data. Second model input are again clinical data with features extracted from a pretrained GoogleNet, on an U-Net segmented area. The third model is an end-to-end deep learning model trained from scratch, with clinical data feed in a multi layer perceptron and then concatenated with CNN in a fully connected layer. In ten fold cross validation, the reported accuracy were 0.755, 0.769 and 0.748, with support vector machine as the best performing machine learning method in both models. They also adopted a leave-one center-out cross validation, with reported accuracy of 0.752, 0.743 and 0.709, respectively, and logistic regression as traditional classifier.

## Methods

### Patients and dataset

The dataset for this retrospective study consists of patients admitted to Fondazione Poliambulanza Istituto Ospedaliero (Brescia, Italy) between February 20, 2020 and May 6, 2020 with confirmed diagnosis of COVID-19. The hospital was at the forefront of fighting the disease outbreak in northern Italy in the first months of 2020. Diagnosis was made via nasopharyngeal swab analyzed through the Reverse Transcriptase-Polymerase Chain Reaction, RT-PCR. Patients with baseline thoracic CT images, arterial blood gas analysis data, total blood counts and Lactate Dehydrogenase test (LDH) were considered for this study. This last has been chosen as inclusion criterion due to his effectiveness as inflammatory biomarker for COVID-19^30,31^. We chose a binary outcome in two severity classes, evaluated at discharge, defined as follows:

1. ICU class: death or intensive care unit admission;
2. Non-ICU class: patients discharged as healed or transferred to non-COVID wards for further care.

**Figure 2:**
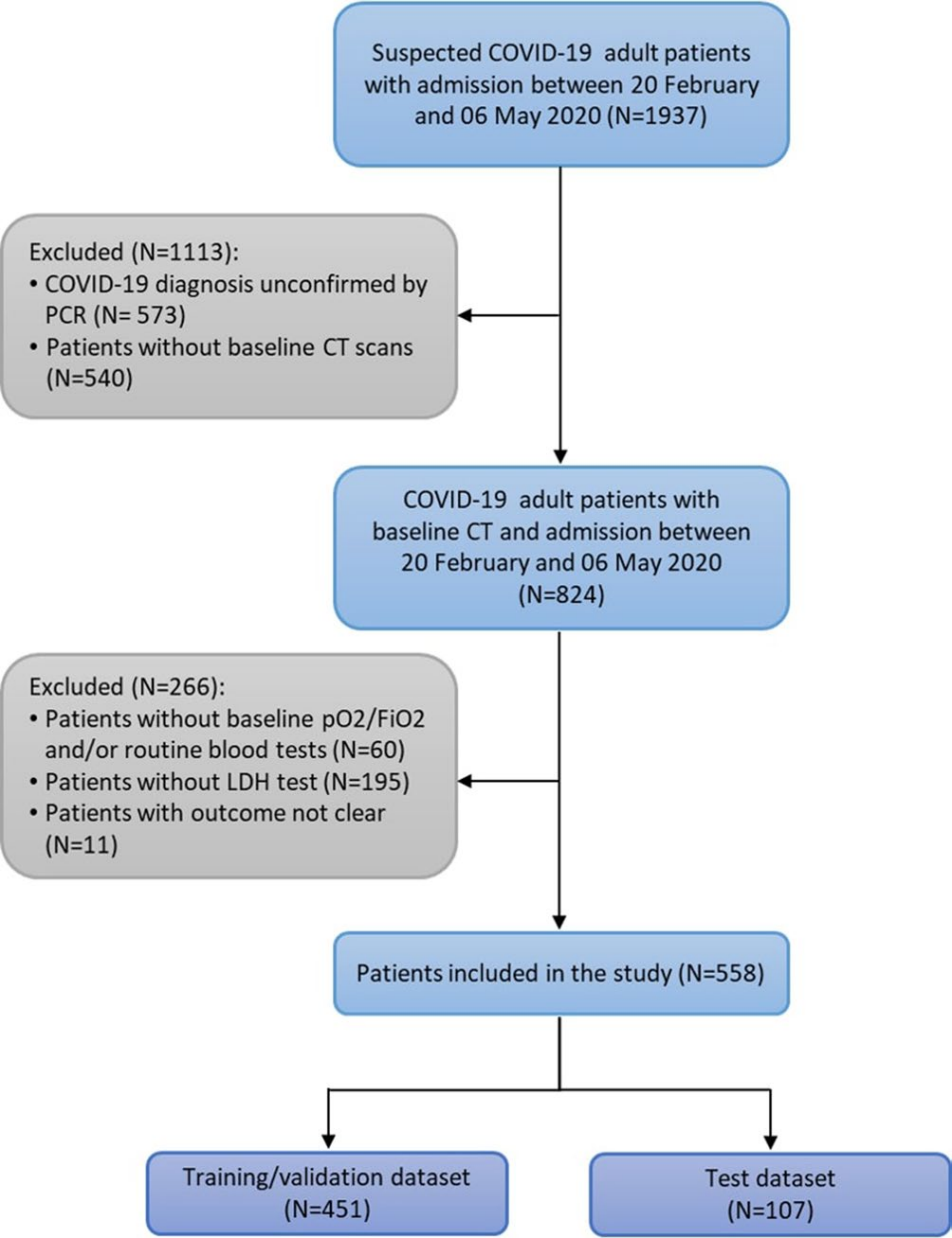
Flowchart of patients inclusion/exclusion.

We excluded patients for which outcome reconstruction was uncertain (e.g. due to early transferral to other hospitals or care structures). A total of 558 patients met these criteria. Figure 2 shows the flowchart of patients selection. Variables missing in more than 20% of cases were excluded, even if their predictive efficacy has been advocated, e.g. D-dimer^32^, Interleuchin-6^33^. Variables obviously redundant were merged (e.g. differential white cells count in percent and absolute values). The 40 variables selected are shown in Table 1. They consist of:

- Anagraphic information (sex and age) and biometric data (Body Mass Index)
- Comorbidities (diabetes, hypertension, cardiovascular disease, oncological condition)
- Vital signs ad admission (Body Temperature, Heart Rate, Blood Pressure)
- Arterial blood gas analysis (${\mathrm{PCO}_2}$, ${\mathrm{HCO}_3}$,${\mathrm{PaO}_2/\mathrm{FiO}_2}$, Lactate, $\mathrm{SO}_2$)
- Complete blood count
- Additional blood /laboratory analysis.

**Figure 3:**
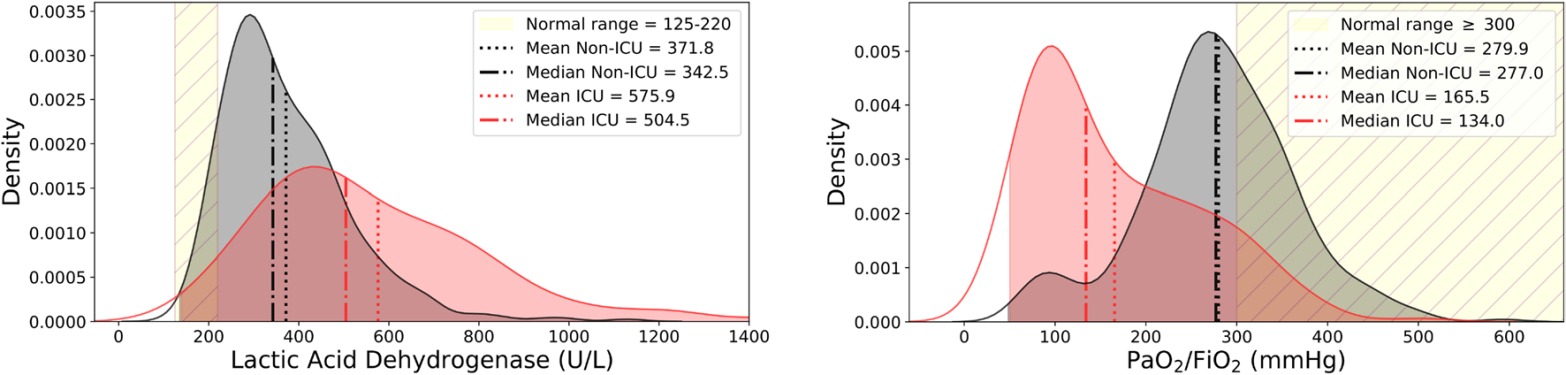
Distributions of Lactic Acid Dehydrogenase and ${{ \mathrm PaO}_2/\mathrm{FiO}_2}$ for patients in Non-ICU (grey) and ICU (red) severity classes. Yellow area is normal value range. Mean and median values are also indicated. LDH is an effective inflammatory biomarker. ${\mathrm{PaO}_2/\mathrm{FiO}_2}$ is a biomarker of lung functionality.

The ${\mathrm{PaO}_2/\mathrm{FiO}_2}$ measures the oxygen saturation level of the patient, allowing to assess its hypoxaemia staus, and therefore the functionality of the lungs. The role of inflammation and immune response is fundamental in the progression of COVID-19 disease. Anomalies in many inflammation biomarkers has been reported in COVID-19 patients^34^. In particular, besides LDH, serum C-reactive protein (CRP), but also alanine aminotransferase (ALT), and aspartate aminotransferase (AST) anomalous levels have been observed. In blood counts, lymphopenia has been observed in up to 83% of hospitalized patients^35^. Figure 3 shows the respective distribution of two prominent biomarkers, LDH and ${\mathrm{PaO}_2/\mathrm{FiO}_2}$ for both outcome classes. Deviations from normality are apparent for both classes.

**Table 1:** Summary of clinical and laboratory variables used.

Binary					
Variable	Total	Non-ICU	ICU		
Sex	F = 166 M = 392	F = 121 M = 259	F = 45 M = 133		
Diabetes	Y = 131 N = 427	Y = 80 N = 300	Y = 51 N = 127		
Hypertension	Y = 255 N = 303	Y = 165 N = 215	Y = 90 N = 88		
Cardiovascular Disease	Y = 263 N = 295	Y = 164 N = 216	Y = 99 N = 79		
Oncological (last 5 yrs)	Y = 41 N = 517	Y = 33 N = 347	Y = 8 N = 170		

Numerical					
Variable	Measure Unit	Median	Median: Non-ICU	Median: ICU	Reference range
Age	yrs	66	64	69	
Body Mass Index (BMI)		26	25.9	26	
Body Temperature	°C	37.5	37.4	37.7	< 37
Heart Rate (HR)	bpm	92	92	90	60–100
Diastolic Blood Pressure (DBP)	mmHg	76	77	75	60–80
Systolic Blood Pressure (SBP)	mmHg	127	127	127	90–120
**Arterial blood gas analysis**					
$\mathrm{pCO}_2$	mmHg	36	35	39	35–48
$\mathrm{HCO}_3$	mmol/L	25.4	25.4	25.4	21–28
$\mathrm{PaO}_2/\mathrm{FiO}_2$	mmHg	255	277	134	$\ge$ 300
Lactate (LAC)	mmol/L	1.1	1.0	1.3	0.5–1.6
SO$_{2}$	%	94	94.5	91.8	95–99
**Complete blood count**					
White Blood Cell Count (WBC)	$\times 10^{9}/L$	7	6.6	8.3	4.5–10
Red Blood Cell Count (RBC)	$\times 10^{12}/L$	4.3	4.4	4.3	4.2–6.3
Hemoglobin (Hb)	g/dL	13.1	13.2	13	14–18
Hematocrit (HCT)	%	39.8	39.8	39.6	40–52
Red Blood Cell Distribution Width (RDW)	%	12.3	12.1	12.6	10.6–13.8
Granulocyte Neutrophils %	%	78	75	84.7	41–70
Granulocyte Eosinophils %	%	0.2	0.2	0.2	1–5
Granulocyte Basophils %	%	0.2	0.3	0.2	0.1–2
Monocytes %	%	6.5	7.4	5	1–12
Lymphocytes %	%	14.2	16.6	9.4	20–50
Platelets (PLT)	$\times 10^{9}/L$	189	198	176	130–450
**Additional blood/laboratory analysis**					
Erythrocyte Sedimentation Rate (ESR)	mm/h	5.5	5.4	5.6	Variable
C-reactive Protein (CRP)	mg/L	92	71	151	< 5
Albumin	g/dL	3.2	3.3	3.2	3.1–5.2
Prothrombin Time International Normalized Ratio (PT INR)		1	1	1.1	0.8–1.2
Aspartate Aminotransferase (AST)	U/L	46	43	55	< 60
Alanine Aminotransferase (ALT)	U/L	34	33	35	< 35
Total Bilirubin	mg/dL	0.7	0.6	0.7	< 1.2
Creatine kinase (CK)	U/L	102	86	163	30–200
Lactic Acid Dehydrogenase (LDH)	U/L	388	343	505	125–220
Sodium	mmol/L	140	140	140	136–145
Potassium	mmol/L	4.1	4.1	4.1	3.3–5.1
Creatinine	mg/dL	0.84	0.8	0.96	0.72–1.18
Urea	mg/dL	38	34	47	18–55

### CT acquisition protocols

Chest CT were acquired using two 64 slices scanners Optima CT 660 (GE Medical Systems, Milwaukee, USA). All patients were examined in supine position. Due to differences in clinical presentation and accession type (i. e. emergency department, general practitioner prescription, incidental finding), one of four different acquisition protocols was used, with differences in slice width, slice spacing and pitch (see Table 2). For all protocols, tube voltage was 120 kVp and automatic current modulation was used. The reconstruction algorithm were mixed filtered back projections-iterative (ASIR). In each protocol, more than one reconstructed volume was available, usually with different proportions of filtered back-projection and iterative algorithm (e.g. lung, bone and parenchyma optimized).

**Table 2:** CT acquisition protocols.

Protocol	#1	#2	#3	#4
% of cases	70%	20%	7%	3%
Transverse resolution (mm)	0.765	0.765	0.765	0.765
Slice width (mm)	2.5	1.25	2.5	2.5
Slice spacing (mm)	2.5	1.25	0.625	1.1
Pitch	1.375	0.969	1.375	0.984

### Model overview

**Figure 4:**
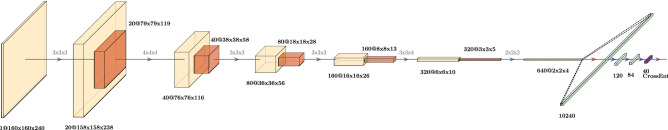
A representation of the CNN architecture used. Actual model is volumetric, i.e. three spatial dimensions plus a channels dimension. Green arrows represent convolution operations with stride of 1. A ReLU nonlinear activation is applied after convolutions, and then a 2×2×2 max pooling in order to reduce spatial dimensions. Red arrow represents flattening. Blue arrows are full connections (with a 0.25 dropout), purple arrow stands for the final classifier with Log SoftMax and Cross Entropy loss function.

The proposed model is composed as follows:

- a fully 3D CNN patient-level classifier on CT images (Fig. 4);
- feature extraction from the last Fully Connected Layer of the CNN;
- a dimensionality reduction procedure including Principal Component Analysis (PCA) on extracted image features, a preliminary CatBoost model and the Boruta algorithm with the SHAP feature importance as metric (BorutaSHAP^36^);
- a CatBoost classifier on the reduced feature space.

The dataset was split in train/validation and test (holdout) subsets, in a 0.8:0.2 proportion (N$_{\mathrm{train/valid}}$ = 451, ICU = 147, non-ICU = 304 and N$_{\mathrm{test}}$ = 107, ICU = 31, non-ICU = 76, respectively; see Supplementary Table S1 for demographic data of the split). Ten fold stratified cross validation was applied in the train/validation set, in order to perform model selection and tuning. The final model obtained was then evaluated on the test. Overall validation strategy is not trivial, due to feature extraction and feature selection steps (see Fig. 6). In brief, for each of the ten folds of the cross validation, CNN was trained on the training set, and evaluated on the validation set. In this way CNN hyperparameters were chosen, in particular the number of epochs. The same training/validation split was used for PCA analysis, to reduce dimensionality of extracted features. On the same training set, a BorutaSHAP feature selection procedure on combined image-extracted (and reduced) and clinical/laboratory features was performed, and also a reduction of the CatBoost hyperparameter space to be searched. The same validation set is then used to pick the best CatBoost hyperparameter choice for the fold. At this point, there were ten different models (feature extraction+ PCA+ BorutaShap+CatBoost), each with its own score on its validation set. The best performing one was considered as the best overall hyperparameter choice, and retrained on the joined training and validation sets, in order to use for training the maximum number of data available without compromising evaluation on the test. The retrained model was applied to the test set for final evaluation. Prediction uncertainty on this final evaluation was estimated with the booststrap method. The output of the model is the percentage score of the classification and the SHAP feature importance values at patient level.

### Image preprocessing

All CT scans were transformed with bicubic interpolation to a common spatial resolution of 1.625 mm $\times$ 1.625 mm $\times$ 2.5 mm. A rigid registration to a single CT picked as representative was performed (6 degrees of freedom, mutual information metric). This step was done in order to minimize small patient positioning differences, and therefore to make easier the following registration-based lung mask creation. Besides, a common pose allows a smaller volume size as input for the CNN. A lung mask was created on the basis of non-rigid method registration of a known CT with lung mask to the target CT^37,38^. Deformable registration was performed with regularized B-spline method, with mutual information metric and GPU acceleration. All registrations (rigid and deformables) were performed with Plastimatch^39^, with default parameters (when not specified). This method of mask creation was successfully applied to lungs affected by severe tuberculosis^40^, where traditional threshold-based and region-growing methods usually fail. It was chosen as a quick and easy to implement lung extraction method, able to deal even with worst pneumonia cases. Once masked images were produced, a volume of size 160$\times$160$\times$240 was obtained with zero-padding. At this point, different reconstructions for the same CT scans were merged (mean values were used), in order to reduce the effect of reconstruction algorithm choice, obtaining one single baseline volumetric image for each patient. Volumes were then z-normalized (mean value was subtracted and the results were divided by standard deviation).

### Tabular missing data

Non-imaging missing data have been replaced with median imputation (i.e., the median value of the feature has been substituted for the missing value). In order to avoid knowledge leakage, median imputation was always performed after test/ validation/ training split (i.e., missing values in the test set has been substituted with median values of the test set, and so on).

### Volumetric convolutional neural network

The first block of the proposed model is a patient-level 3D CNN classifier, with six convolutional layers with ReLU activation followed by max pooling, and three fully connected layers with a 0.25 dropout, plus a final classification layer. The loss function is CrossEntropy. Group normalization is used, due to its better efficacy with small batches^41^. In practice, in a generic CNN normalization procedure, from each feature computed by a layer, a mean value is subtracted and the result is divided by standard deviation, where mean and standard deviation are computed along a subset of indices. In the most commonly used batch normalization mean and standard deviation are computed on spatial and batch indexes, for each channel. When batches are smalls, a more effective normalization procedure would be layer normalization, where mean and standard deviation are computed along spatial and channel indices, for each batch element^42^. Group normalization is a refinement of layer normalization, in which channel space is divided in groups, and mean and standard deviation are computed on spatial and channel indexes of each group. In this way, through an hyperparameter (the number of groups) it is possible to have a finer control on the strength of the normalization, at the same time preserving a larger part of channels diversity (and therefore information). The CNN block is shown in Fig. 4.

### CNN training and data augmentation

Data augmentation was performed in each fold on the fly, only for each training set, in the ten cross validation folds. Data augmentation techniques used were:

- Affine deformation. During every epoch, there was a 50% of probability to apply a random affine deformation with rotation between 0 to 10 degrees and a size variation up to 10%.
- Elastic deformation. A random displacement was attributed to a grid of $7\times 7\times 7$ control points assigned to every images, with a maximum displacement equals to 10 voxels in each direction along cartesian axes. The displacement at every voxel was interpolated using a cubic B-spline function.

All the techniques were implemented using the framework Torchio^43^. Training was performed with the Stochastic Gradient Descent (SGD) optimizer and a fixed learning rate of $3\times 10^{-5}$. The number of epochs was chosen for each training/validation fold on the basis of AUC result on the validation set (the best in a fixed number of 50). For each fold, features at the input of the final classification layer were extracted (40 features).

### Principal component analysis

Principal Component Analysis (PCA) was used on features extracted, in order to reduce the dimensionality from 40 to 5 features. The usage of PCA to provide an out of the box, unsupervised, dimensionality reduction for CNN extracted features has been already proven effective in hybrid approaches^44^. In this work we applied PCA only to CNN extracted image features, that can be considered agnostic, while the subsequent feature selection preserves interpretability.

### Feature selection: BorutaSHAP overview

A feature selection procedure was performed with BorutaSHAP and a preliminary CatBoost classifier, on the 40 non-imaging features and the 5 imaging features from PCA. The Boruta algorithm is an all relevant feature selection method, i.e. it tries to select all the features relevant for a given ensemble model. Relevance is evaluated against shadow features, that is dummy features created from real ones with random reordering of values^45^. In the BorutaSHAP Python implementation, features and shadow features are compared by means of their SHAP importance values, producing therefore a result more consistent than other metrics^46,47^. The level of feature elimination can be tuned via a (percentile based) multiplicative factor on maximum shadow feature.

### Feature selection: classifier choice

As every other wrapper method, BorutaSHAP needs a classifier (to evaluate features and shadow features importance). As classifier, we trained a preliminary CatBoost model (on the training subset), using the whole 45 features. In order to obtain a quicker convergence, the preliminary model has a fixed tree number (700) and a learning rate at double of automatic CatBoost suggestion. For other hyperparameters, Bayesian optimization was performed with the automatic optimization framework Optuna 2.3.0^48^, with 300 trials (0.8:0.2 calculation/evaluation split), on the training set only.

### Feature selection: nested voting procedure

**Figure 5:**
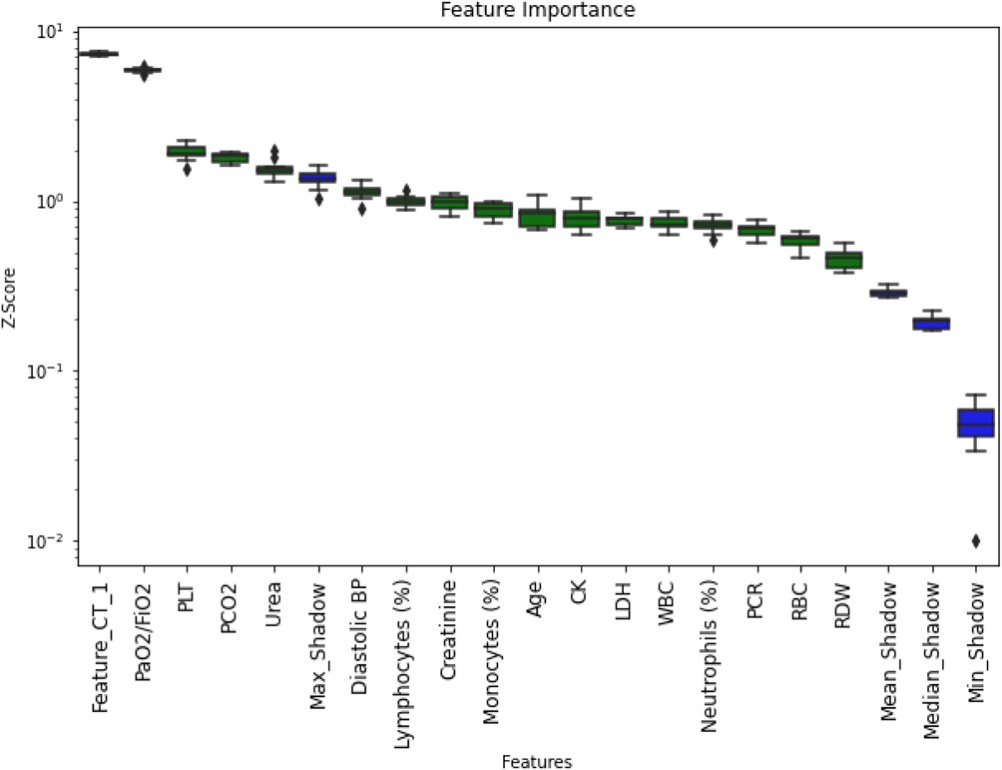
A representative BorutaSHAP importance plot. Green are features to keep in the model for this fold. Blue are maximum, mean, median and minimum shadow features.

**Figure 6:**
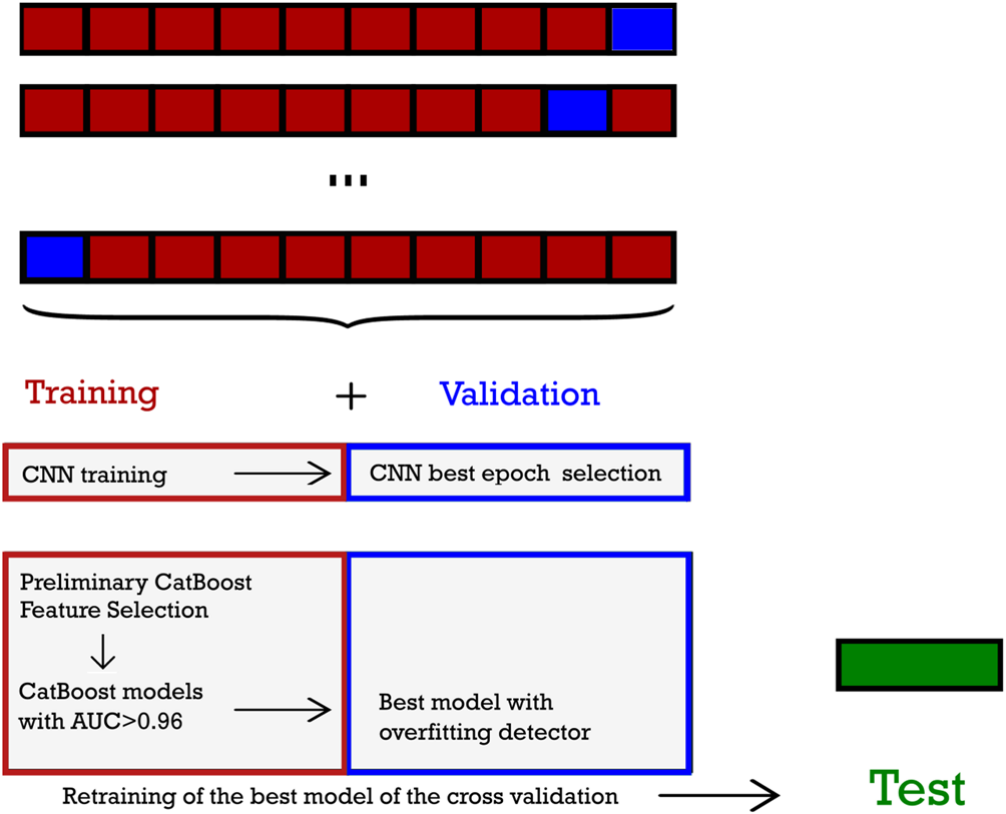
A sketch of the cross validation procedure with feature selection. The dataset is split in test, used only for final evaluation, and training/validation, used for CNN training and evaluation, deep learned feature extraction, feature selection and hyperparameter tuning. Ten fold cross validation is applied in the training/validation set. CNN is trained on the training set (upper left red box), evaluated for hyperparameters on the validation set (upper right blue box). Extracted features are combined with non-imaging features, and selected in the training set, with a preliminary model (lower left red box: Preliminary CatBoost+Feature Selection). Bayesian optimization with Optuna is used for the preliminary model hyperparameters choice. Feature selection is effected with BorutaSHAP. CatBoost hyperparameters tuning on the selected feature set was effected in two steps, first with abayesian optimization in order to reduce the hyperparameter (lower left red box: CatBoost models with AUC$>0.96$) and then with overfitting detector (lower right blue box: best model in validation set). The best model of cross validation is retrained on the combined training/validation set, and evaluated on the test.

In our dataset, SHAP feature importance tends to have a slowly degrading distribution, except for the two most important features (CT first principal component and PaO$_{2}$/FiO$_{2}$; an example is shown in Fig. 5). Unfortunately, such small difference in feature importance could result in an inherent hypersensitivity of feature selection to small changes in the dataset, with consequent lack of generalization power. In particular, we perceived that elimination of an important feature was a worst eventuality than keeping a feature of scarce importance. In order to increase the robustness of feature selection and minimize the risk of leaving out an important feature, we implemented a nested majority voting feature elimination strategy. In other words, we repeated the BorutaSHAP feature selection 8 times with random patients reordering, each time with 7 parts of the training set used for the wrapper model and the eighth to compare features and shadow features importance. In this way we got eight choices of features. A feature absent in six over eight choices was eliminated. Note that the whole feature selection procedure was applied for each of the ten training subsets, keeping the same splits used for the CNN (Fig. 6).

### CatBoost model

We built a CatBoost classifier on the reduced feature set, keeping the same training and validation splits used for CNN, with a two steps procedure for hyperparameters optimization:

- Selection of a reduced number of hyperparameter combinations (the best performing on the training set), with the aid of Bayesian optimization, at fixed learning rate and number of trees.
- The selected combinations were compared on the validation set, with a fixed learning rate and a number of trees optimized by the overfitting detector.

The best model was chosen by AUC on its validation set. It was then retrained on the joined training and validation subset, with a 120% number of trees in order to keep in account the larger training size. Such final model was evaluated on the test/holdout dataset. A graphical resume of the cross validation and testing procedure is shown in Fig. 6. The rationale of the procedure is to control the computational burden of hyperparameter search, and at the same time to fully exploit the potential of the overfitting detector for number of trees selection by means of early stopping. In the first step, Bayesian optimization in the training set was performed with the Optuna optimizer, with parameters as in the previous subsection. For the first step, learning rate was fixed at the values automatically calculated by CatBoost on the basis of the number of instances and features. Models with AUC $\ge$ 0.96 were selected for validation testing (an empirically chosen threshold value). In the second step, learning rate was fixed at a constant value of 0.008 (at the lower end of the range of values for the first step). The number of trees was picked with the CatBoost overfitting detector as the best performing on the validation subset, starting with a very large value, 20000. In this way, almost complete freedom is left to the overfitting detector to stop at the best iteration. In practice the final model has fixed learning rate, a Bayesian-optimized combination of hyperparameters, and a number of trees selected by the overfitting detector. Hyperparameters of the final CatBoost model are reported in Supplementary Table S2. The final CatBoost model is used for prediction on the test set. Note that up to this final evaluation, the test set was never involved at any point of the procedure, in order to avoid any data leakage. Confidence intervals on the results on test set were evaluated with the bootstrap method on the test set.

### Implementation and code availability

The overall model implementation has been made in Python 3.7 with open source libraries. In particular the framework PyTorch 1.7^49^ has been used for the CNN block. The PC utilized for the training is equipped with a Intel^®^ Core^TM^ i7-8700 CPU (6 cores, 12 threads, 3.2 GHz) and a NVIDIA^®^ GeForce^®^ RTX 2080 Ti GPU (11 GB memory). The code is available at https://github.com/matteochieregato/GradientboostingCovid19.

### Ethical aspects

The study has been approved by the ethical committee of Brescia (Comitato Etico di Brescia: protocol number NP 4274 STUDIO GBDLCovid, session of 06/04/2021). All methods were carried out in accordance with relevant guidelines and regulations. The aforementioned protocol regulated informed consent collection and authorized its waiving where not possible (for patients not traceable or because they abandoned the center).

## Results

### CNN results

**Figure 7:**
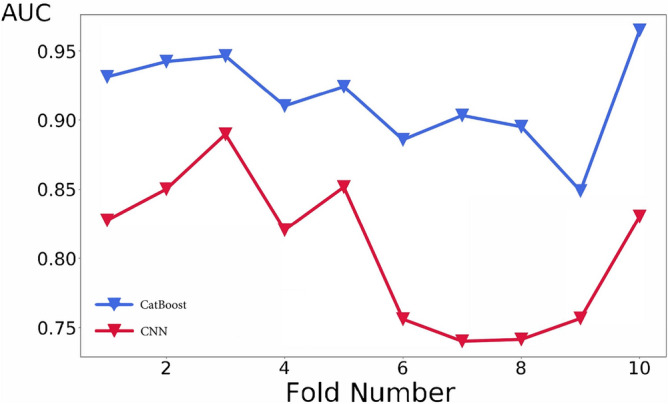
Cross validation of the CatBoost and CNN classifiers. A roughly common trend can be discerned, however the highest score is reached at different folds (3rd for the CNN and 10th for the CatBoost classifier).

Results of the CNN classifier in terms of AUC is shown in Fig. 7 for the ten validation subsets. The third validation fold has the best AUC score, 0.889 (mean AUC in the ten folds is 0.806). Variation in CNN results are likely due to a combination of causes. First, in our fully 3D model, each instance corresponds to a patient, and therefore the number of instances used for the training (330) is not so large for deep learning, even with data augmentation. Second, establishing a prognostic model on the basis of CT imaging alone is possibly a difficult task, at least on our dataset. The time passed between the manifestation of the first symptoms and presentation at hospital (and therefore CT acquisition) was highly variable, due to the grievous situation at the time, with overburdened hospitals. Experience told us that the progression of COVID-19 can be very fast. It is possible that imaging alone for a number of cases in our dataset simply is not sufficient for meaningful prognostic predictions.

### CatBoost predictive power

**Figure 8:**
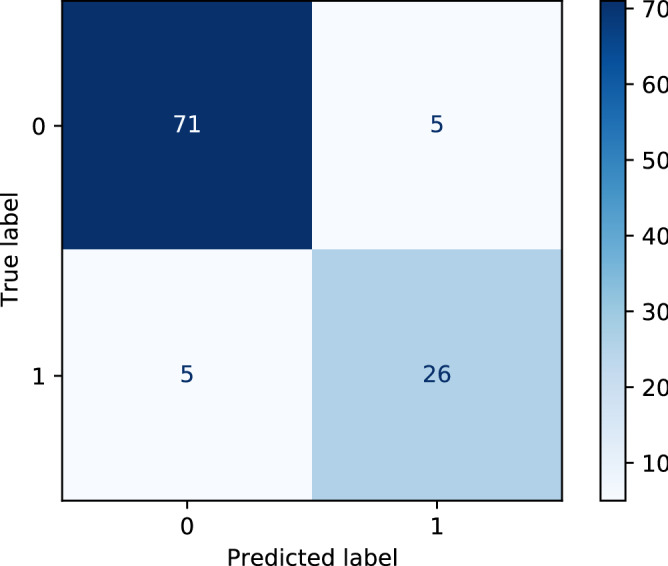
Confusion matrix obtained with the best model on the test set (0 : non-ICU patients and 1: ICU patients).

AUC classification results of CatBoost is shown in Fig. 7 for each of the ten validation subsets (mean AUC = 0.915). The final best model reaches AUC = 0.949 in the test set, with a 95% confidence interval of 0.899–0.986. The confidence interval is calculated with the bootstrap method with 10000 folds resampling of the test set. Figure 8 shows the confusion matrix for the test set (Sensitivity = 83.9%, Specificity = 93.4%). Since the model is intended as probabilistic classifier, it is optimized on probabilistic AUC, not on sensitivity and specificity. Setting the threshold for ICU prediction at 0.25 instead of 0.5, sensitivity becomes 90.3% with a specificity of 89.5%.

### Feature selection and global level feature importance

**Figure 9:**
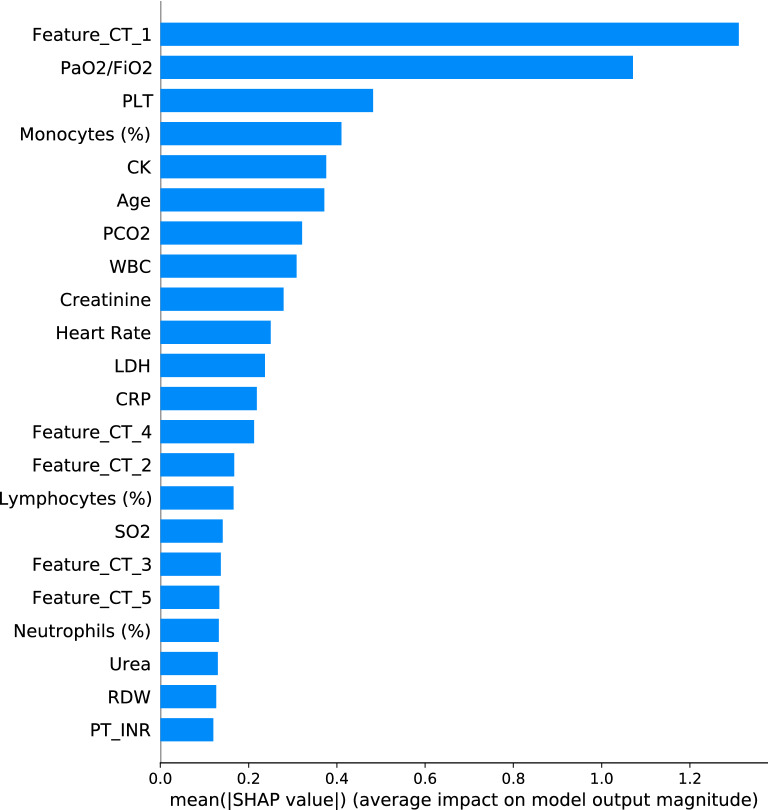
Mean absolute value of the SHAP values for each feature in the test set.

Figure 9 shows the 22 features selected by our procedure in the best model, along with SHAP global feature importance in prediction over the test set. The first CT principal component and the $\mathrm{PaO}_{2}/\mathrm{FiO}_{2}$ stand out.

### Patient level feature importance

**Figure 10:**
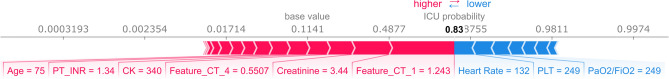
Force plot of SHAP values for a single patient. Less important features are omitted for the sake of visualization. Features are represented as arrows that push the outcome (black small vertical line), either towards a ICU outcome (red arrows) or a non-ICU outcome (blue arrows). The black number over the small vertical line, 0.83, is the probability of the outcome for this patient. The length of the arrows is proportional to the SHAP values of the associated features for this particular prediction. Under each arrow it is reported the corresponding feature name and value. More details in the text.

Figure 10 shows a force plot of the SHAP output feature importance for a single patient prediction^10^. In this graph, features are represented as forces (arrows) that push the outcome prediction (the black bar with a bold number over it) in positive or negative direction. The length of the arrow is proportional to its Shapley value. The color of the arrow corresponds to positive or negative influence of the feature. Keeping the game metaphor used in the Introduction, we could consider the red and blue features as two teams, pushing the black bar in opposite direction. Features are team players, and the Shapley value of each features is represented as the strength of the player. Here ICU outcome is red, and non-ICU is blue. The case shown is correctly predicted as ICU with an 83% score. We can see that in this case, CT features (1 and 4), creatinine, creatine kinase, prothrombin time and old age all push the prediction to an ICU outcome. On the other side, the value of $\mathrm{PaO}_{2}/\mathrm{FiO}_{2}$ is better than most ICU cases (249, corresponding to mild Acute Respiratory Distress Syndrome^50^). Also heart rate and platelet value contribute negatively to the prediction score, i.e. they push toward a non-ICU outcome.

### Model introspection

We analyzed on a case-by-case basis the patients for which the final model gave a wrong prediction, in particular ICU outcomes misclassified as non-ICU. It turned out that for 2 out of such 5 patients in the test set, there were meaningful additional information not taken in account by the model. In one case, there was a full scale D-dimer value (well known as indicator of poor outcome^32^). In the other, the patient is insulin-dependent type 1 diabetic. Diabetes comorbidity was eliminated by the feature selection procedure. Indeed, in our dataset a specific type 1 effect could have been hidden by the overwhelming majority of type 2 diabetic patients. Such cases highlight the supporting role for which the proposed model is rightly intended for. Note that if these two cases were to be excluded, sensitivity would be 89.7%.

## Discussion

### Imaging and non-imaging data combination

Complex tasks in clinic need integration of radiological information with laboratory and clinical information. Machine learning methods are starting to be employed for such a purpose. Besides COVID-19 prognosis, examples can be Alzheimer disease classification and progress^51^ or the individuation of immunotherapy responders^52^.

Radiological information is native as imaging data, while laboratory and clinical information comes in tabular form. Up to now, there is still no consensus on the best way to combine these two types of data in machine learning models. In particular, CNN are showing “unreasonable effectiveness” in image related task^53,54^ in the last years. However, the same is simply not true for tabular data^55^, where ensemble models, and especially gradient boosting variations (XGBoost^56^, LightGBM^57^, CatBoost^7^), have the edge^58,59^. Efforts to build deep learning models dedicated to tabular data (e.g. NODE^60^, TabNet^61^) have shown remarkable results in some dataset, but weaker performance in other, despite considerable complexity^59^.

In principle, an integration of imaging and non-imaging information that harnesses the power of neural network in a combined model can be reached in a number of ways. Essentially, they boil down to four types:

1. neural network for segmentation only
2. combination of results of separate imaging and tabular models
3. tabular data injection in a deep learning model
4. extraction of learned image features and construction of a combined tabular data model.

The first and perhaps the simplest of these approaches is the use of CNN only for segmentation. On such basis, various quantification indices and/or handcrafted features can be calculated and fed in a tabular model. For COVID-19 prognosis, this is the approach of^16–19^. Another simple method is to combine the results of a deep learning classifier on images with either clinical/laboratory features or the independent results of a tabular model^20–24^. Both these strategies reached remarkable results. However, neither of the two pushes towards truly integration of information in machine learning. In both, imaging models and tabular models are kept separated, and interaction between features of the two domains is neglected (in the “combining the results” approach), or demanded to handcrafted features (in the “segmentation” approach). The other two methods truly aim to build a combined model, in which information is fused at a lower level, so to allow a full interaction between different domains. In the tabular injection approach, non imaging data are concatenated at some points of a deep learning classifier, with a fully connected layer being the obvious choice. Tabular data can be injected as they are, or after elaboration, for example after one or more fully connected layer. This method allows to build fully differentiable models, end-to-end trainable. As such, it is also easier to validate. In COVID-19 prognosis, this method is used by^25,26,62^, and in the third model of^29^. Remarkable examples in other fields are^63^ for Alzheimer diagnosis,^64^ for Alzheimer’s converters early detection,^65^ for skin lesions classification. The fourth approach is to use CNN as image feature extractor and a different machine learning model on the top to operate on both image extracted and non image features on equal footing (e.g.^27^, the second model by^29^). Note that CNN can be pretrained (as in^29^) or trained from scratch (as in^27^ and in the present work). This method has several advantages. It can exploit a state-of-art model for heterogeneous data (e.g. gradient boosting^66–68^ for extracted CNN features in XGBoost classifiers). The underlying machine learning architecture is less prone to data-starving, it can be naturally integrated with advanced feature selection algorithms, and it is more readily explainable once agnostic features for images are accepted as such, since its symmetrical elaboration of non-imaging and image extracted features.

Our dataset consists of few hundred of patients, a small number for CNN applications. Prognosis is a patient-level task, and as such we believe that number of patients, not of CT slices, is the fundamental number of instances. Furthermore, there is a perceived need for explainability of artificial intelligence applications, especially in the clinics (see below). Therefore, we chose to sacrifice full differentiability and opted for the fourth method.

### Model building and training

For COVID-19 prognosis, global features are likely to be more effective than spatially localized features (that could be more useful for diagnosis in initial phases). Therefore a fully 3D patient-level architecture is the more appropriate choice for the task. A CNN classifier allows to pick the high level representation features relevant to the task. At the end of the network, a multiple fully connected layer structure allowed us a gradual reduction of the number of features before their extraction, so to balance it with non-imaging features. CatBoost was used as the machine learning classifier for the final model. CatBoost is becoming increasingly applied in complex datasets^69^. It implements Ordered Boosting, a permutation driven version of boosting algorithm, and Oblivious Decision Trees, a particular type of decision trees (as well as other features we do not treat here). Both should be especially effective in avoiding overfitting. Hancock and Khoshgoftaar^69^ pointed out that CatBoost performance is likely sensitive to hyperparameters choice. We especially picked by hand some hyperparameters (Ordered Boosting as boosting type and Bayesian bootstrap type) so to select the solution less prone to overfitting, using Bayesian optimization for most of the others. The most influential hyperparameters are the learning rate and the number of trees. For these, CatBoost provides very powerful tuning methods, respectively with the automatic learning rate estimate and the overfitting detector, and we made use of both. The feature selection in our model is based on the combination of the Boruta algorithm with the SHAP metric, as implemented by Keany et al.^36^. The Boruta algorithm tries to find all relevant features for the task (and the model), not a compact subset that minimize information loss for the classifier^45^. The use of the SHAP metric naturally keeps in account feature interactions and cooperative effects. We implemented a majority voting procedure in order to exploit the strengths of BorutaSHAP, at the same time minimizing the risk of information loss and the dependence of subsampling randomness (Subsection CatBoost model). Since validation set is used as such both for CNN feature extractor and CatBoost hyperparameter choice, we can not completely exclude that some knowledge leaks from the feature extraction along our dimensionality reduction procedure up to the hyperparameter choice. We believe that our selected procedure, in particular the restriction of feature selection and Bayesian hyperparameter optimization on the training set should minimize the impact of knowledge leakage (and therefore the risk of overfitting). In any case no leakage on the test set was possible, due to holdout from the start.

### Model interpretability

There is a general debate about the need of interpretability of machine learning models for decision making^70^. Notably, European Union legislation assesses the right to have an explanation of a decision made after automated data processing (GDPR16^71^). We believe that an even stronger push for model explainability comes from clinical needs. In particular, an explainable model is not only more acceptable for doctors and patients, but becomes much more integrable with additional, out-of-the-model information (see Subsection Model introspection). In the proposed model, interpretability at global level and especially as single prediction level is given by the SHAP analysis. CT features, being extracted from the CNN classifier and the PCA reduction, are agnostic. However, one can still use them to appraise the overall weight of CT both in general and single case predictions. In particular, the first principal component is much more significant than the others, so it can be used as a proxy.

### Limitations

There are limitations to the proposed model. First, the dataset comes from a single center, in a localized period of time, with consequent trade-off between data homogeneity and generalization power. Second, the number of our patients is limited in comparison to the usual numbers in deep learning classification tasks. Larger datasets naturally tend to reduce model variance. To reduce the influence of these two limitations, we took particular care in trying to avoid overfitting.

Finally, any endpoint for COVID-19 related task can be potentially influenced by the pressure posed to hospitals by the large numbers of patients e.g. mortality rate and/or choice of admission to intensive care units can change. We considered an ICU admission severity outcome to be more applicable in clinical context than a mortality prediction. However, we are aware that such an outcome definition is calibrated on our center (i.e. a different center can have different admission criteria to intensive care unit). We believe that the choice of an interpretable, probabilistic output can reduce the bias due to outcome choice.

## Conclusion

We built a COVID-19 prognostic hybrid machine-learning/deep learning model intended to be usable as a tool that can support clinical decision making. The proposed model fully integrates imaging and non-imaging data. A 3D CNN classifier extracts patient level features from baseline CT scans. A CatBoost classifier is applied on extracted features and laboratory and clinical data. Feature selection in the model is performed via the Boruta algorithm combined with the SHAP feature importance. Such architecture blends state-of-art machine learning for tabular data with the efficacy of a 3D CNN in building and selecting patient-level complex image features. The tool is interpretable at global and at single patient level, with the SHAP importance of features in obtaining the percentage score of classification. Such analytical result is susceptible to be integrated by ulterior information that the clinician may have. We think that at the present state of things, this is the correct clinical usage of machine learning for COVID-19 prognostic tasks. There is a certain number of COVID-19 prognostic models that make use of radiological and clinical data with deep learning techniques. However, only an handful of them are truly integrated models built on heterogeneous features. The proposed model follows this approach, in line with^25–27,29,62^. As such, it allows models to take into account feature interactions. In particular an high degree of interaction between heterogeneous features is expected for COVID-19 prognosis task, due to complex relations between anatomical and functional lung involvement and systemic inflammatory response.

In our knowledge, the present work is the first published one to use CatBoost on top of deep learning extracted features. It is also the first work to apply a gradient boosting model on combined CNN extracted features and clinical and laboratory data to COVID-19 prognosis. The proposed model was trained on a limited size dataset, without image segmentation from the radiologists. It would be therefore easily retrainable from scratch in order to adapt it to the mutable landscape of the pandemic, due to different variants of the virus, differences in the affected population demographics and effects of vaccine campaigns. Efforts in artificial intelligence triggered by the pandemic are likely to pave the way to future applications in different clinical contexts. We believe that the integration of heterogeneous data and the interpretability of models will be keypoints for any clinical application involving complex tasks.

## Supplementary Information

Supplementary Information.

## Data Availability

The dataset analyzed during the current study can be made available from the corresponding author on reasonable requests upon ethical committee approval.
