# Supplementary material for: A hybrid machine learning/deep learning COVID-19 severity predictive model from CT images and clinical data
Source: Sci Rep. 2022 Mar 14;12:4329. doi: 10.1038/s41598-022-07890-1 (PMC8919158; doi:10.1038/s41598-022-07890-1)
Supplement: Supplementary file 1 — Supplementary Information. [file 41598_2022_7890_MOESM1_ESM.pdf]

Table S1. Non imaging variables in the train/validation and test datasets

**Binary**				
Variable		Train/validation	Test	
Sex		F=134 M=317	F=32 M=75	
Diabetes		Y=107 N=344	Y=24 N=83	
Hypertension		Y=199 N=252	Y=56 N=51	
Cardiovascular Disease		Y=222 N=229	Y=41 N=66	
Oncological (last 5 yrs)		Y=34 N=417	Y=7 N=100	
**Numerical**				
Variable	Measure Unit	Median (10th-90th PCTL): Train/validation	Median (10th-90th PCTL): Test	Reference range
Age	yrs	66 (50-80)	64 (46.2-76.8)	
Body Mass Index (BMI)		25.7 (21.7-32.1)	27.2 (23.4-33.3)	
Body Temperature	°C	37.5 (36.4-38.9)	37.5 (36.3-38.8)	< 37
Heart Rate (HR)	bpm	92 (68-116)	91 (74-118)	60-100
Diastolic Blood Pressure (DBP)	mmHg	76 (63-88)	76 (64-89)	60-80
Systolic Blood Pressure (SBP)	mmHg	127 (107-158)	129 (106-155)	90-120
Arterial Blood Gas Analysis				
pCO_2	mmHg	36 (29-48)	36 (31-43)	35-48
HCO_3	mmol/L	25.4 (20.8-29.7)	25.5 (22.2-29.1)	21-28
PaO_2/FiO_2	mmHg	254 (89-356)	260 (86-378)	≥ 300
Lactate (LAC)	mmol/L	1.1 (0.6-2.2)	1.0 (0.6-2.1)	0.5-1.6
SO_2	%	94.1 (82.5-98.1)	93.5 (80.7-98)	95-99
Complete Blood Count				
White Blood Cell Count (WBC)	$\cdot 10^9/L$	7 (3.6-13.8)	7.17 (3.8-12.8)	4.5-10
Red Blood Cell Count (RBC)	$\cdot 10^{12}/L$	4.3 (3.6-5)	4.4 (3.8-5)	4.2-6.3
Hemoglobin (Hb)	g/dL	13 (10.8-14.8)	13.3 (11.2-14.7)	14-18
Hematocrit (HCT)	%	39.7 (33.5-45.4)	39.9 (34.3-45.6)	40-52
Red Blood Cell Distribution Width (RDW)	%	12.3 (11.5-14)	12.0 (11.2-13.5)	10.6-13.8
Granulocyte Neutrophils %	%	78 (57.4-90.3)	77.5 (54.6-89.3)	41-70
Granulocyte Eosinophils %	%	0.2 (0.1-1.6)	0.2 (0.1-1.5)	1-5
Granulocyte Basophils %	%	0.2 (0-0.9)	0.2 (0-0.9)	0.1-2
Monocytes %	%	6.4 (2.8-11.5)	6.7 (3.5-11.9)	1-12
Lymphocytes %	%	14.1 (5.4-30.4)	14.2 (5.8-31.8)	20-50
Platelets (PLT)	$\cdot 10^9/L$	181 (107-335)	204 (126-309)	130-450
Additional Blood / Laboratory Analysis				
Erythrocyte Sedimentation Rate (ESR)	mm/hour	5.5 (1.9-8.1)	5.4 (1.9-8)	*variable*
C-reactive Protein (CRP)	mg/L	94.3 (17-246.9)	82.7 (19.1-229)	< 5
Albumin	g/dL	3.2 (2.7-3.7)	3.2 (2.8-3.6)	3.1-5.2
Prothrombin Time International Normalized Ratio (PT INR)		1.02 (0.92-1.27)	1 (0.93-1.19)	0.8-1.2
Aspartate Aminotransferase (AST)	U/L	46 (25-101)	48 (23-103)	< 60
Alanine Aminotransferase (ALT)	U/L	33 (13-92)	38 (14-107)	< 35
Total Bilirubin	mg/dL	0.7 (0.5-1.3)	0.6 (0.4-1.2)	< 1.2
Creatine kinase (CK)	U/L	102 (33-498)	100 (36-363)	30-200
Lactic Acid Dehydrogenase (LDH)	U/L	391 (229-699)	375 (242-699)	125-220
Sodium	mmol/L	140 (135-144)	140 (136-145)	136-145
Potassium	mmol/L	4.1 (3.4-4.9)	4 (3.5-4.9)	3.3-5.1
Creatinine	mg/dL	0.85 (0.64-1.6)	0.83 (0.68-1.6)	0.72-1.18
Urea	mg/dL	38 (21-82)	38 (24-90)	18-55

Table S2. CatBoost main hyperparameters in the final trained model

Hyperparameter	Value
Loss function	LogLoss
Iterations/number of trees	12483
Learning rate	0.008
L2 leaf regularization term	4.136
Boosting type	Ordered
Bootstrap type	Bayesian
random subspace method	0.076
Bagging temperature	3.102
